# 1877. Post-Tuberculosis Treatment Mortality in Georgia (2009-2018)

**DOI:** 10.1093/ofid/ofad500.1705

**Published:** 2023-11-27

**Authors:** Sarah Gorvetzian, Antonio Pacheco, Erin Anderson, Susan M Ray, Marcos Schechter

**Affiliations:** University of Colorado, Denver, Colorado; Fundaçāo Oswaldo Cruz, Rio de Jainero, Rio de Janeiro, Brazil; Georgia Department of Public Health, Atlanta, Georgia; Emory University School of Medicine, Atlanta, Georgia; Emory University School of Medicine, Atlanta, Georgia

## Abstract

**Background:**

There is growing evidence that people with tuberculosis (TB) are at higher risk of mortality even after treatment completion. However, there are limited data on post-TB treatment mortality rates and causes of death in the United States (U.S.).

**Methods:**

Cohort study of all adults (age ≥ 18 years) diagnosed with TB in Georgia (U.S.) who finished treatment between January 1, 2008, and December 31, 2019. We obtained post-TB treatment mortality data from the Centers for Disease Control and Prevention National Death Index and general Georgia population death rates and causes of death for the same period from public databases. We calculated age- and sex-standardized mortality rates (SMRs) for the TB cohort and general Georgia population and the SMR ratio between these two groups (along 95% confidence interval estimates).

**Figure:**
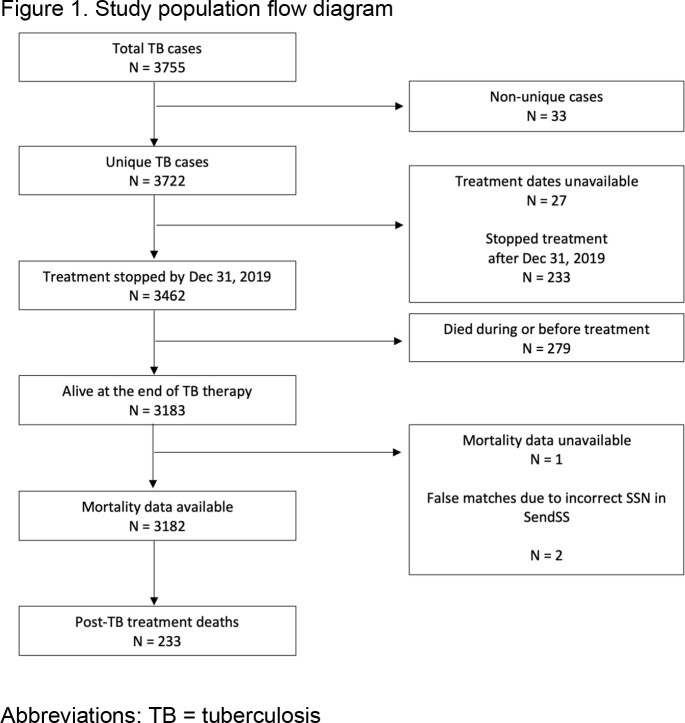

**Results:**

Among 3,183 patients alive at the time TB treatment was stopped, 2,391 (75.1%) were culture-confirmed, 625 (19.6%) were extrapulmonary, and 15 (< 1%) were rifampin and isoniazid resistant. The median age at TB diagnosis was 44 years (interquartile range (IQR): 32-57), 2,094 (65.8%) were male, and 1,557 (48.9%) were U.S.-born. 328 (10.3%) were co-infected with HIV. Most patients (n=2,973, 93.4%) completed treatment. Reasons for treatment non-completion included adverse events (n=11), patient declined treatment (n=13), and lost to follow-up (n=186). The median post-TB treatment follow-up was 6.4 years (IQR 3.4-9.0) and 233 (7.3%) patients died. Causes of death included cardiovascular disease and malignancy (n=56 or 24%, each) and HIV (n=22, 9.9%). The age and sex adjusted SMR ratio of the TB cohort compared to the general Georgia population was 0.93 (95% CI: 0.78-1.09). The SMR ratio in a subgroup analysis restricted to U.S.-born persons was 1.6 (95% CI: 1.36-1.77).

**Table 1.**
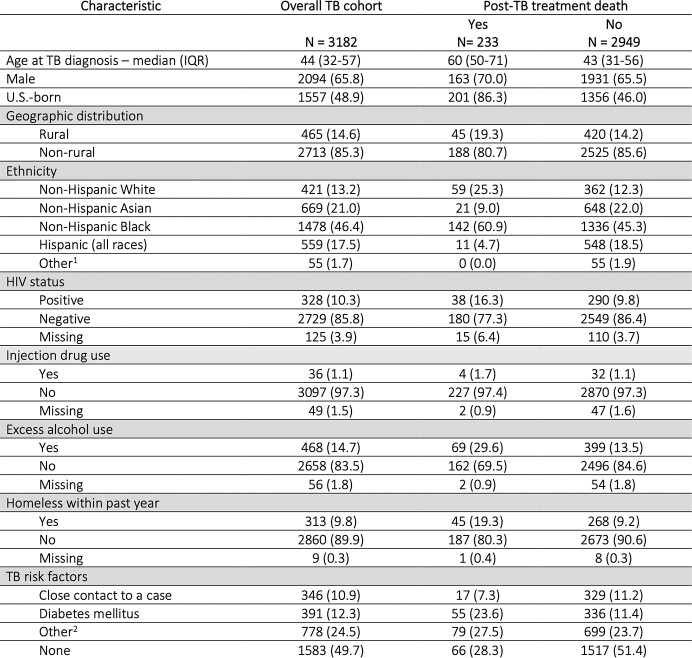
Baseline demographic characteristics

All data is shown as n (%) unless stated otherwise. Abbreviations: TB = tuberculosis, IQR = inter-quartile range 1. Other races = American Indian/Alaska Native (total n = 3, death post treatment n = 0), multiracial (total n = 11, death post treatment = 0) 2. Other risk factors = end-stage renal disease (total n = 43, death post-TB treatment n = 9), post-organ transplantation (total n = 7, death post-TB treatment n = 1), TNF-a antagonist therapy (total n = 8, death post-TB treatment n = 0), immunosuppression (not HIV/AIDS) (total n = 75, death post-TB treatment n = 8), incomplete LTBI (total n = 112, death post-TB treatment n = 7), other (total n = 533, death post-TB treatment n = 54); missing data (total n = 368, death post-TB treatment n = 47)

**Table 2.**
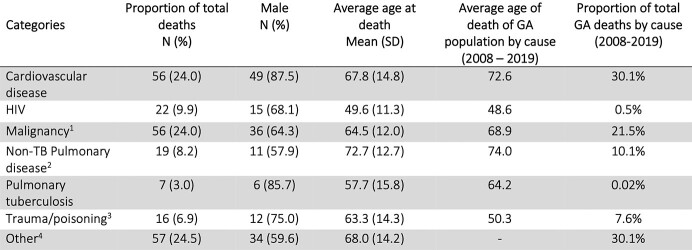
Causes of death post-TB treatment and in the general Georgia population. Abbreviations: TB = tuberculosis, SD = standard deviation, GA = Georgia 1. Pulmonary malignancy: n = 21 (37.5% of total malignancies, 9.0% of total deaths) 2. Pulmonary TB/mycobacterial infection: n = 7 3. Poisoning due to narcotics or hallucinogens: n = 2 (0.86%); proportion of total GA deaths for disorders related to drug use = 0.43% 4. Alcohol-related liver disease: n = 3 (1.3%); proportion of total GA deaths due to alcohol-related liver disease

**Table 3.**
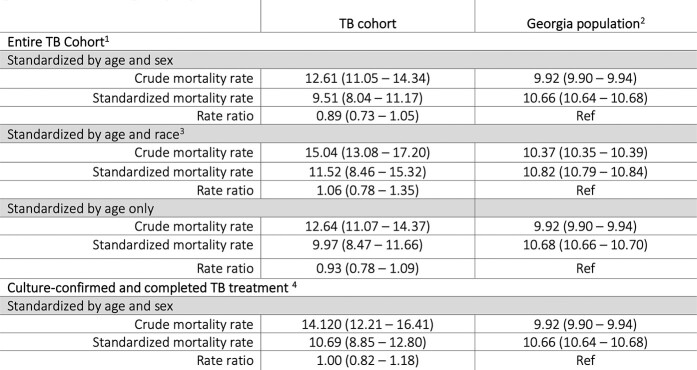
Tuberculosis cohort standardized mortality rates and rate ratios compared to the general Georgia population

All rates are per 1000, (95% confidence interval). Abbreviations: TB = tuberculosis 1. Entire TB cohort: deaths n = 233, person-time = 18,440 person-years 2. Georgia population: deaths n = 900,874, person-time = 90,796,252 person-years 3. Race includes only non-Hispanic White and non-Hispanic Black categories: TB cohort (race standardization): deaths n = 212, person-time = 14,095 person-years GA population (race standardization): deaths n = 888,261, person-time = 85,630,800 person-years 4. Culture-confirmed TB cohort: deaths n = 182, person-time = 12,821 person-years

**Conclusion:**

Our data suggests the overall cohort of patients who finish TB treatment are not at higher risk of death compared to the general population. Analysis restricted to U.S.-born persons, however, did show a slightly higher risk of death post-TB treatment, which suggests the so-called "healthy immigrant effect" could have affected our post-TB treatment mortality estimates.

**Disclosures:**

**All Authors**: No reported disclosures

